# Supplementary material for: Accuracy of the Geriatric Depression Scale (GDS)-4 and GDS-5 for the screening of depression among older adults: A systematic review and meta-analysis
Source: PLoS One. 2021 Jul 1;16(7):e0253899. doi: 10.1371/journal.pone.0253899 (PMC8248624; doi:10.1371/journal.pone.0253899)
Supplement: S3 Fig — (DOCX) [file pone.0253899.s004.docx]

## S3 Fig. Cheng version

| Cheng (cut-off 1)    † ≥75 years, ‡ 60 – 74 years  Cheng 2004 was evaluated in two different groups (Sample A (N=310), Sample B (N=134)) |
| --- |
| Cheng (cut-off 2)    † ≥75 years, ‡ 60 – 74 years  Cheng 2004 was evaluated in two different groups (Sample A (N=310), Sample B (N=134) |
| Cheng (cut-off 3)    † ≥75 years, ‡ 60 – 74 years  Cheng 2004 was evaluated in two different groups (Sample A (N=310), Sample B (N=134)) |
| Sensitivity of Cheng (cut-off 4)    Cheng 2004 was evaluated in two different groups (Sample A (N=310), Sample B (N=134)) |
| Specificity of Cheng (cut-off 4)    Cheng 2004 was evaluated in two different groups (Sample A (N=310), Sample B (N=134)) |
