# Supplementary material for: Accuracy of the Geriatric Depression Scale (GDS)-4 and GDS-5 for the screening of depression among older adults: A systematic review and meta-analysis
Source: PLoS One. 2021 Jul 1;16(7):e0253899. doi: 10.1371/journal.pone.0253899 (PMC8248624; doi:10.1371/journal.pone.0253899)
Supplement: S4 Fig — (DOCX) [file pone.0253899.s005.docx]

## S4 Fig. Galaria version

| Sensitivity of Galaria (cut-off 2)   |
| --- |
| Specificity of Galaria (cut-off 2)   |
