# Supplementary material for: Accuracy of the Geriatric Depression Scale (GDS)-4 and GDS-5 for the screening of depression among older adults: A systematic review and meta-analysis
Source: PLoS One. 2021 Jul 1;16(7):e0253899. doi: 10.1371/journal.pone.0253899 (PMC8248624; doi:10.1371/journal.pone.0253899)
Supplement: S5 Fig — (DOCX) [file pone.0253899.s006.docx]

## S5 Fig. Hoyl version

| Hoyl (cut-off 2)  ****  † Non-demented  In Rinaldi 2003, we considered the reported data of all patients evaluated. |
| --- |
