# Supplementary material for: Accuracy of the Geriatric Depression Scale (GDS)-4 and GDS-5 for the screening of depression among older adults: A systematic review and meta-analysis
Source: PLoS One. 2021 Jul 1;16(7):e0253899. doi: 10.1371/journal.pone.0253899 (PMC8248624; doi:10.1371/journal.pone.0253899)
Supplement: S6 Fig — (DOCX) [file pone.0253899.s007.docx]

## S6 Fig. Cheng or Heisel version and De Dios or Ortega version

| Sensitivity of Heisel or Cheng (cut-off 1)    † 60 – 74 years, ‡ ≥75 years |
| --- |
| Specificity of Heisel or Cheng (cut-off 1)    † 60 – 74 years, ‡ ≥75 years |
| Sensitivity of Heisel or Cheng and De Dios or Ortega (cut-off 2)    † 60 – 74 years, ‡ ≥75 years |
| Specificity of Heisel or Cheng and De Dios or Ortega (cut-off 2)    † 60 – 74 years, ‡ ≥75 years |
| Sensitivity of Heisel or Cheng (cut-off 3)    † 60 – 74 years, ‡ ≥75 years |
| Specificity of Heisel or Cheng (cut-off 3)    † 60 – 74 years, ‡ ≥75 years |
| Sensitivity of Heisel or Cheng (cut-off 4)    † 60 – 74 years, ‡ ≥75 years |
| Sensitivity of Heisel or Cheng (cut-off 4)    † 60 – 74 years, ‡ ≥75 years |
