# Supplementary material for: Accuracy of the Geriatric Depression Scale (GDS)-4 and GDS-5 for the screening of depression among older adults: A systematic review and meta-analysis
Source: PLoS One. 2021 Jul 1;16(7):e0253899. doi: 10.1371/journal.pone.0253899 (PMC8248624; doi:10.1371/journal.pone.0253899)
Supplement: S1 Table — (DOCX) [file pone.0253899.s008.docx]

## S1 Table. Search strategy

| Database | Search strategy | Date of Search | Results |
| --- | --- | --- | --- |
| Pubmed | (“Geriatric Depression Scale”[tiab] OR “5-GDS”[tiab] OR “4-GDS”[tiab] OR “GDS-4”[tiab] OR “GDS-5”[tiab] OR “Yesavage”[tiab] OR ((“4-item”[tiab] OR “4-items”[tiab] OR “5-item”[tiab] OR “5-items”[tiab]) AND GDS[tiab])) AND (sensitiv*[TIAB] OR specificity[TIAB] OR "Predictive Value of Tests"[Mesh] OR "sensitivity and specificity"[MeSH] OR diagnosis[MeSH] OR Cronbach*[TIAB] OR ROC[TIAB] OR AUC[TIAB] OR “Area under”[TIAB]) | **April 24^th^, 2020** | 1,385 |
| PsycINFO | (TI Geriatric Depression Scale OR AB Geriatric Depression Scale OR TI 5-GDS OR AB 5-GDS OR TI 4-GDS OR AB 4-GDS OR TI GDS-4 OR AB GDS-4 OR TI GDS-5 OR AB GDS-5 OR TI Yesavage OR AB Yesavage OR ((TI “4-item” OR AB “4-item” OR TI “4-items” OR AB “4-items” OR TI “5-item” OR AB “5-item” OR TI “5-items” OR AB “5-items”) AND (TI GDS OR AB GDS))) AND (TI sensitiv OR AB sensitiv OR TI specificity OR AB specificity OR DE “sensitivity and specificity” OR MJ “sensitivity and specificity” OR DE diagnosis OR MJ diagnosis OR MA diagnostic OR MJ diagnostic OR TI Cronbach* OR AB Cronbach* OR TI ROC OR AB ROC OR TI AUC OR AB AUC OR TI “Area under” OR AB “Area under”) | **April 24^th^, 2020** | 386 |
| Scopus | ( TITLE-ABS-KEY ( "Geriatric Depression Scale" OR "5-GDS" OR "4-GDS" OR "GDS-4" OR "GDS-5" OR "Yesavage" ) OR ( TITLE-ABS-KEY ( "4-item" OR "4-items" OR "5-item" OR "5-items" ) AND TITLE-ABS-KEY ( GDS ) ) ) AND TITLE-ABS-KEY ( sensitiv* OR specificity OR Cronbach* OR ROC OR AUC OR “Area under” ) | **April 24^th^, 2020** | 869 |
| Google Scholar | geriatric depression scale sensitivity specificity | **May 16^th^, 2020** | First 100 |
