# Supplementary material for: Accuracy of the Geriatric Depression Scale (GDS)-4 and GDS-5 for the screening of depression among older adults: A systematic review and meta-analysis
Source: PLoS One. 2021 Jul 1;16(7):e0253899. doi: 10.1371/journal.pone.0253899 (PMC8248624; doi:10.1371/journal.pone.0253899)
Supplement: S2 Table — (DOCX) [file pone.0253899.s009.docx]

## S2 Table. Excluded studies

| N° | Author, Year | Title | Reason for exclusion |
| --- | --- | --- | --- |
| 1 | Amadori, 2011 | Comparison of the 15-item geriatric depression scale (GDS-15) and the GDS-4 during screening for depression in an in-patient geriatric patient group | Letter to the editor |
| 2 | Flacker, 2003 | Does question comprehension limit the utility of the geriatric depression scale in older African Americans? | Letter to the editor |
| 3 | Bonin-Guillaume, 1995 | [Psychometric evaluation of depression in the elderly subject: which instruments? What are the future perspectives?] | Review |
| 4 | Edwards, 2004 | Assessing for depression and mood disturbance in later life | Review |
| 5 | Grossberg, 2017 | Rapid Depression Assessment in Geriatric Patients | Review |
| 6 | Scogin, 2006 | Screening older adults for depression in primary care settings | Review |
| 7 | Seymour, 2005 | Validation of short screening tests for depression: Comment on Goring et al. (2004) | Letter to the editor |
| 8 | Bass, 2008 | An efficient screening tool for preoperative depression: the Geriatric Depression Scale-Short Form | GDS-4 or GDS-5 were not assessed |
| 9 | Fujisawa, 2005 | The development of a brief screening instrument for depression and suicidal ideation for elderly: the Depression and Suicide Screen | GDS-4 or GDS-5 were not assessed |
| 10 | Gerety, 1994 | Performance of case-finding tools for depression in the nursing home: influence of clinical and functional characteristics and selection of optimal threshold scores | GDS-4 or GDS-5 were not assessed |
| 11 | Hsiao, 2015 | Development of the psychometric property of a Minimum Data-Set-Based Depression Rating Scale for use in long-term care facilities in Taiwan | GDS-4 or GDS-5 were not assessed |
| 12 | Kadhim, 2018 | Validating screening tools for depression in Parkinson's disease | GDS-4 or GDS-5 were not assessed |
| 13 | Kim, 2010 | Depressive symptoms in elderly adults with hypotension: different associations with positive and negative affect | GDS-4 or GDS-5 were not assessed |
| 14 | Lam, 2004 | Depression in dementia: a comparative and validation study of four brief scales in the elderly Chinese | GDS-4 or GDS-5 were not assessed |
| 15 | Lelito, 2001 | Psychometric evaluation of a brief geriatric depression screen | GDS-4 or GDS-5 were not assessed |
| 16 | Liu, 1998 | Correlations between scores on Chinese versions of long and short forms of the Geriatric Depression Scale among elderly Chinese | GDS-4 or GDS-5 were not assessed |
| 17 | McCurren, 2002 | Assessment for depression among nursing home elders: evaluation of the MDS mood assessment. Geriatric nursing | GDS-4 or GDS-5 were not assessed |
| 18 | Min, 2015 | Validation of the K6/K10 Scales of Psychological Distress and Their Optimal Cutoff Scores for Older Koreans | GDS-4 or GDS-5 were not assessed |
| 19 | Mui, 1996 | Geriatric Depression Scale as a community screening instrument for elderly Chinese immigrants | GDS-4 or GDS-5 were not assessed |
| 20 | Saracino, 2017 | Assessing depression in a geriatric cancer population | GDS-4 or GDS-5 were not assessed |
| 21 | Tomaszewski, 2011 | [Validation of the Patient Health Questionnaire-9 Polish version in the hospitalised elderly population] | GDS-4 or GDS-5 were not assessed |
| 22 | Wong, 2002 | Development and inter-rater reliability of a standardized verbal instruction manual for the Chinese Geriatric Depression Scale-short form | GDS-4 or GDS-5 were not assessed |
| 23 | Zalsman, 1998 | Geriatric depression scale-short form-validity and reliability of the hebrew version | GDS-4 or GDS-5 were not assessed |
| 24 | Chang, 2011 | The Collateral Source version of the Geriatric Depression Scale: evaluation of psychometric properties and discrepancy between collateral sources and patients with dementia in reporting depression | Accuracy was not assessed |
| 25 | Chau, 2006 | Factor structure of the Chinese version of the Geriatric Depression Scale | Accuracy was not assessed |
| 26 | Diaz, 2011 | Reliability and construct validity of MUNSH test to measure happiness, in elderly munsh chilean population | Accuracy was not assessed |
| 27 | Hallit, 2017 | Validation of the Arabic Geriatric Depression Scale (GDS-5) among the lebanese geriatric population | Accuracy was not assessed |
| 28 | Hammami, 2012 | [Screening for depression in an elderly population living at home. Interest of the Mini-Geriatric Depression Scale] | Accuracy was not assessed |
| 29 | Lucas-Carrasco, 2012 | Spanish version of the Geriatric Depression Scale: reliability and validity in persons with mild-moderate dementia | Accuracy was not assessed |
| 30 | Parashos, 2002 | Recognition of depressive symptoms in the elderly: what can help the patient and the doctor | Accuracy was not assessed |
| 31 | Song, 2014 | Depression as a correlate of functional status of community-dwelling older adults: utilizing a short-version of 5-item Geriatric Depression Scale as a screening tool | Accuracy was not assessed |
| 32 | Clement, 1997 | Development and contribution to the validation of a short French version of the Geriatric Depression Scale | Unable to retrieve |
| 33 | Hegamin-Younger, 1999 | Use of the Iowa Self-Assessment Inventory with older hospitalized patients | Unable to retrieve |
| 34 | Kurlowicz, 1999 | The Geriatric Depression Scale (GDS) | Unable to retrieve |
| 35 | Lach, 2010 | Can older adults with dementia accurately report depression using brief forms? Reliability and validity of the Geriatric Depression Scale | Unable to retrieve |
| 36 | Lee, 1994 | Cross-validation of the Geriatric Depression Scale short form in the Hong Kong elderly | Unable to retrieve |
| 37 | Marquez, 2006 | Validation of geriatric depression scale-5 scores among sedentary older adults | Not enough data for analysis |
| 38 | Santos, 2019 | Validation study of a reduced version of the geriatric depression scale in Portugal | Not enough data for analysis |
| 39 | D'Ath, 1994 | Screening, detection and management of depression in elderly primary care attenders. I: The acceptability and performance of the 15 item Geriatric Depression Scale (GDS15) and the development of short versions. | Used other reference standards |
| 40 | Shah, 1996 | Screening for depression among geriatric inpatients with short versions of the geriatric depression scale | Used other reference standards |
| 41 | Shah, 1997 | Screening for depression among acutely ill geriatric inpatients with a short Geriatric Depression Scale | Used other reference standards |
| 42 | Hoyl, 2000 | Depresión en el adulto mayor: evaluación preliminar de la efectividad, como instrumento de tamizaje, de la versión de 5 ítems de la Escala de Depresión Geriátrica | Used other reference standards |
| 43 | Isella, 2002 | Screening and Quantification of Depression in Mild-to-Moderate Dementia Through the GDS Short Forms | Used other reference standards |
| 44 | Weeks, 2003 | Comparing various short-form Geriatric Depression Scales leads to the GDS-5/15 | Used other reference standards |
| 45 | Goring, 2004 | Validation of short screening tests for depression and cognitive impairment in older medically ill inpatients | Used other reference standards |
| 46 | Cully, 2005 | A 2-item screen for depression in rehabilitation inpatients | Used other reference standards |
| 47 | Storandt, 2005 | Use of the 5-Item Geriatric Depression Scale in Demented Individuals and with Psychiatric Outpatients | Used other reference standards |
| 48 | Nguyen, 2006 | Screening for depression in hospitalised and community-dwelling elderly: the use of the 4-item, 5-item and 15-item geriatric depression scales | Used other reference standards |
| 49 | Park, 2006 | Comparing Various Short-Form Geriatric Depression Scales in Elderly Patients | Used other reference standards |
| 50 | Li, 2015 | Validity of the geriatric depression scale and the collateral source version of the geriatric depression scale in nursing homes | Used other reference standards |
| 51 | Korner, 2006 | The Geriatric Depression Scale and the Cornell Scale for Depression in Dementia. A validity study | Not specified GDS-4 or GDS-5 items |
| 52 | Clement, 1999 | Detection of depression in elderly hospitalized patients in emergency wards in France using the CES-D and the mini-GDS: preliminary experiences | Not specified GDS-4 or GDS-5 items |
