# Supplementary material for: Accuracy of the Geriatric Depression Scale (GDS)-4 and GDS-5 for the screening of depression among older adults: A systematic review and meta-analysis
Source: PLoS One. 2021 Jul 1;16(7):e0253899. doi: 10.1371/journal.pone.0253899 (PMC8248624; doi:10.1371/journal.pone.0253899)
Supplement: S3 Table — (DOCX) [file pone.0253899.s010.docx]

## S3 Table. Characteristics of the included studies

| **Author (Country), Year** | **Setting** | **N** | **Population and inclusion/exclusion criteria** | **Female sex** | **Age (mean)** | **Index test** | **Language of the test** | **Mode of test assesment** | **Reference standard** | **Prevalence of depression** | **Funding** |
| --- | --- | --- | --- | --- | --- | --- | --- | --- | --- | --- | --- |
| Van Marwijk (Netherlands), 1995 | Clinic outpatients | 586 | Aged ≥ 65 years (no cognitively impaired, able to read or write, understanding the procedure, no too depressed, no too ill, no language problems) | 59.6% | 76.3 | GDS-4 by Van Marwijk | Dutch | Self-administered | Major depression and dysthymia assessed by DIS based on DSM-III | 5.63% | Self-funded |
| Almeida (Brazil), 1999 | Clinic outpatients | 64 | Aged ≥ 60 years (no severe sensory impairment, no aphasia, Mini-Mental State score ≥ 10) | 84.4% | 67.45 | GDS-4 by Van Marwijk | Portuguese | Face-to-face interview | Major depressive episode (F32) and dysthymia (F34.1) assessed by ICD-10 checklist of symptoms according to ICD-10, and DSM-IV | ICD-10: 51.6%  DSM-IV: 64.1% | Brazilian Research Counceil |
| Hoyl (US), 1999 | Clinic outpatients | 74 | Aged ≥ 65 years with frailty (no lack of geropsychiatric evaluation or no unclear psychiatric diagnosis) | 1.4% | 74.6 | GDS-4 by D’Ath, Van Marwijk and GDS-5 by Hoyl | English | Face-to-face and telephone interview | Major depression and depression not otherwise specified assessed by PRIME-MD based on DSM-IV | 46% | Self-funded |
| Galaria (US), 2000 | Clinic outpatients | 70 | Aged ≥ 65 years cognitively intact | 58.6% | 77.4 | GDS-4 by Galaria | English | Face-to-face interview | Major depression assessed by DSM-III-R | 38.57% | Not reported |
| Chattat (Italy), 2001 | Clinic outpatients | 126 | Aged ≥ 53 years | 63.5% | 77.2 | GDS-5 by Hoyl | Italian | Face-to-face interview | Clinical Diagnosis of Depression assessed by DSM (Not specified) | 31.7% | Not reported |
| De Dios (Spain), 2001 | Clinic outpatients | 155 | Aged ≥ 65 years (no MMSE <20,no diseases preventing reliable communication) | 69.7% | 78.3 | GDS-5 by De Dios or Ortega | Spanish | Face-to-face interview | Major depression, dysthymic disorder, an adaptative disorder with depressive mood and adaptative disorder mixed anxious-depressive assessed by DSM-IV | 56.1% | Not reported |
| Pomeroy (UK), 2001 | Clinic inpatients | 87 | Aged ≥ 60 years, (no illness, no communication problems, no 10-item Abbreviated Mental Test <6, no cognitive impairment) | 60% | 78.4 | GDS-4 by D'Ath | English | Face-to-face interview | A depressive episode (F32) assessed by ICD-10 | 19.5% | Self-funded |
| Rinaldi (Italy), 2003 | Clinic outpatients and nursing home patients | 181 | Aged ≥65 years with normal cognitive functions (no cognitive impairment, no severe life-threatening diseases) | Female: 66.85%  Male: 33.15% | 79.4 | GDS-5 by Hoyl | Not detailed | Face-to-face interview | Major depression, dysthymia, bipolar depression, and depression not otherwise specified assessed by DSM-IV | 48.1% | Not reported |
| Cheng (China), 2004 and Cheng, 2005 | Clinic outpatients | 444 / 442 | Aged ≥ 60 years, MMSE > 20, with a one-year period for psychiatric evaluation (no language difficulty, no 4 or more missing items on GDS-30) | Sample A: 61.9% (n= 310)  Sample B: 67.9% (n= 134)  /  63.6% | Sample A: 70.06  Sample B: 69.83  /  69.99 | GDS-4 by Cheng | Chinese | Face-to-face interview | Major depressive disorder, dysthymia, depressive disorder not otherwise specified, adjustment disorder with depressed mood, dementia with depression assessed by DSM-III / Major depression, dysthymia, depressive disorder not otherwise specified, adjustment disorder with depressed mood and dementia with depression assessed by DSM-III-R | Sample A: 41.8%  Sample B: 46.5%  /  Old demented 24.59%(n=61), Old non-demented 48.9%(n=47), Young demented 35.1%(n=111), and young non-demented 54.75%(n=223) | Self-funded / Not reported |
| Jongenelis (Netherlands), 2005 | Nursing homes patients | 333 | Aged ≥ 55 years (MMSE ≥15, no short admission) | 69% | 79.3 | GDS-4 by D'Ath, Van Marwijk and GDS-5 by Hoyl | Dutch | Face-to-face interview | Major depression and minor depression assessed by SCAN for DSM-IV | 22% | Netherlands Organization for Scientific Research |
| Martinez (Spain), 2005 | Clinic outpatients | 249 | Aged ≥ 65 years (without or not a previous diagnosis of depression, no severe psychiatric diagnosis not related to depression, no physical limitations) | 67.5% | 74.3 | GDS-4 by Martinez and GDS-5 by Martinez | Spanish | Face-to-face interview | Clinic diagnosis of depression (Not specified) assessed by ICD-10 | 36.1% | semFYC foundation |
| De la Torre (Peru), 2006 | Clinic outpatients | 400 | Aged ≥ 60 years (no treatment, no cognitive impairment, no psychiatric condition, no moderate to severe cognitive impairment according to Pfeiffer) | 66.25% | 70.85 | GDS-4 by Galaria | Spanish | Face-to-face interview | Clinic diagnosis of depression assessed by DSM-IV | 17.3% | Not reported |
| Castelo (Brazil), 2007 and Castelo, 2010 | Clinic outpatients | 220 | Aged ≥ 60 years and one-year period at four primary care centers | 72.7% | 68.3 | GDS-4 by D'Ath, Van Marwijk | Portuguese | Face-to-face interview | Major depressive episode assessed by SCID-I for DSM-IV | 14% / 17.3% | Ministry of Health/ DECIT/ SESA, Ceara Brazil |
| Izal (Spain), 2007 | Community inhabitants and nursing home patients | 233 | Aged ≥ 60 years, no suspicious of cognitive impairment | 68.7% | 74.1 | GDS-5 by Hoyl | Spanish | Face-to-face interview | Major depression assessed by SCID-I for DSM-IV-TR | 20.17% | Not reported |
| Ortega (Spain), 2007 | Clinic outpatients | 301 | Aged ≥64 years that had consulted at least two times ( no MMSE (30-Item Lobo’s Spanish version) <20, no communication issues) | 57.8% | 74.3 | GDS-5 by De Dios or Ortega | Spanish | Face-to-face interview | Clinical diagnostic of mood disorder assessed by DSM-IV | 14.6% | Ministry of Health and Consumption, the Carlos III Health Institute, the G03 / 170 Research Network on Preventive Activities and Health Promotion in Primary Care |
| Cheng (China), 2010 | Clinic outpatients | 150 | Aged 60-74 years and ≥ 75 years (first time at a geriatric psychiatric outpatient clinic, no MMSE <24) | 58.7% | 74.1 | GDS-4 by Cheng and GDS-5 by Cheng or Heisel | Cantonese | Face-to-face interview | Major depression (F32), dysthymia (F34.1), bipolar disorder-depressive episode (F31.3), adjustment disorder with depressed reactions (F43.21), mixed anxiety and depressive disorder (F41.8), dementia with depressive symptoms (F06.31), assessed by ICD-10 | - Young older group (n=85) 60% - Old-older adult 63.5% (total=65) | Self-funded |
| Izal (Spain), 2010 | Community inhabitants | 105 | Aged ≥ 60 years | 58.1% | 72.9 | GDS-5 by Hoyl | Spanish | Face-to-face interview | Major depression assessed by DSM-IV | 8.6% | Self-funded |
| Allgaier (Germany), 2011 and Allgaier, 2013 | Nursing homes patients | 92 | Aged ≥ 65 years (capability of answering questions, no severely limited cognitive ability or no dementia) | 73.9% | 86.3 | GDS-4 by D'Ath | German | Self-administered | Major depression disorder/ major depression and minor depression assessed by SCID-I for DSM-IV | 14.1% / 28.3% | Self-funded |
| Chin (China), 2014 | Community inhabitants | 388 | Aged ≥ 65 years | 54.4% | 74.3 | GDS-4 by D'Ath, Van Marwijk and GDS-5 by Hoyl, Molloy | Chinese | Face-to-face interview | Major depressive disorder assessed by mPDA according to DSM-III-R | 20.6% | Not reported |
| Apostolo (Portugal), 2018 | Clinic outpatients and inpatients, community inhabitants and nursing home patients | 139 | Aged ≥ 65 years and score ≤21 on the six-item cognitive Impairment test | 60% | 77.68 | GDS-5 by Apostolo | Portuguese | Face-to-face interview | Major depressive episode assessed by DSM-V | 16.5% | Not reported |
| Dokuzlar (Turkey), 2018 | Community inhabitants | 437 | Aged ≥ 65 years (no cognitive deficiency, no delirium psychotic disorder, no acute disease within the last two weeks, no drugs that influence emotional state, no alcohol, no substance addictive) | 63.6% | 72.95 | GDS-4 by Van Marwijk and GDS-5 by Hoyl | Turkish | Face-to-face interview | Major depression assessed by DSM-V | 24.3% | Self-funded |
| Eriksen (Norway), 2019 | Clinic outpatients, community inhabitants, and nursing patients | 194 | Aged ≥ 60 years (no severe aphasia, no life-threatening medical condition) | 74% | 73.4 | GDS-5 by Hoyl | Not detailed | Self-administered | A depressive episode (F32) assessed by ICD-10 | 28.9% | Self-funded |
| Sacuiu (Sweden), 2019 | Clinic outpatients and inpatients, community inhabitants and nursing home patients | 60 | Aged ≥ 65 years and attempt of suicide (no terminal illness, no severe dementia, sufficient knowledge of the Swedish language) | 53.3% | 80.0† | GDS-4 by D'Ath, Van Marwijk and GDS-5 by Hoyl, Cheng or Heisel | Sweden | Face-to-face interview | Major Depressive Disorder assessed by DSM-IV | 28.3% | The Swedish government and the county councils |

DSM: Diagnostic and Statistical Manual of Mental Disorders, ICD: International Classification of Diseases, PRIME-MD: Primary Care Evaluation of Mental Disorders, SCID-I: Structured Clinical Interview for DSM Axis I, DIS: Diagnostic Interview Schedule, PDCdAD: Provisional Diagnostic Criteria for Depression in Alzheimer Disease, BAS-DEP: Brief Assessment Schedule Depression Scale, GMS: Geriatric Mental Status Schedule, SCAN: Schedule of Clinical Assessment in Neuropsychiatry, mPDA: Modified Psychiatrist Diagnostic Assessment, MMSE: Mini-Mental State Examination
