# Supplementary material for: Accuracy of the Geriatric Depression Scale (GDS)-4 and GDS-5 for the screening of depression among older adults: A systematic review and meta-analysis
Source: PLoS One. 2021 Jul 1;16(7):e0253899. doi: 10.1371/journal.pone.0253899 (PMC8248624; doi:10.1371/journal.pone.0253899)
Supplement: Attachment — Submitted filename: Response to Reviewers.docx [file pone.0253899.s014.docx]

**PONE-D-21-03547R1**
**Title:** Accuracy of the Geriatric Depression Scale (GDS)-4 and GDS-5 for the screening of depression among older adults: a systematic review and meta-analysis

**Response letter**

Dear editor and reviewers,

Thank you for your kind consideration. In this letter, we proceed to respond to each of the comments made by the reviewers and the journal requirements.

Sincerely,

Alvaro Taype-Rondan, corresponding author

**Journal Requirements:**

**1.** ***Please review your reference list to ensure that it is complete and correct. If you have cited papers that have been retracted, please include the rationale for doing so in the manuscript text, or remove these references and replace them with relevant current references. Any changes to the reference list should be mentioned in the rebuttal letter that accompanies your revised manuscript. If you need to cite a retracted article, indicate the article’s retracted status in the References list and also include a citation and full reference for the retraction notice.***

**Answer.** Thank you for your observation. We have reviewed the list of references and made changes to the reference style to make it conform to the journal's requirements. We found that Cheng 2004 (1) and Tsoi 2017 (2) have been corrected. We added their “Erratum” information in the references (Lines 383-384 and Line 420).

The list of references is complete, and no retracted studies have been cited.

According to the corrected information from Cheng 2004 (1), we have changed in the column “Reference standard” of Table 1: “Major depressive disorders, dysthymia, adjustment disorder with depressed mood, dementia with depression, bipolar disorder, depressive episode assessed by DSM-III” to “Major depressive disorder, dysthymia, depressive disorder not otherwise specified, adjustment disorder with depressed mood, dementia with depression assessed by DSM-III”. This change was also made in the Supplementary File S3_Table. No other changes to the manuscript were required.

1. Cheng ST, Chan AC. A brief version of the geriatric depression scale for the chinese. Psychol Assess. 2004 Jun;16(2):182-6. doi: 10.1037/1040-3590.16.2.182. Erratum in: Psychol Assess. 2006 Mar;18(1):48. PMID: 15222814.
2. Tsoi KK, Chan JY, Hirai HW, Wong SY. Comparison of diagnostic performance of Two-Question Screen and 15 depression screening instruments for older adults: systematic review and meta-analysis. Br J Psychiatry. 2017;210(4):255-60. doi: 10.1192/bjp.bp.116.186932. Erratum in: Br J Psychiatry. 2017;211(2):120. pmid: 28209592.

**Reviewers' comments:**

**R2.1: *ABSTRACT***

***Methods section:***

***" We conducted sensitivity and specificity meta-analyses of those studies that used.....". Please revise to we conducted meta-analyses of the sensitivity and specificity of those studies that used......***

**Answer.** Thank you for your recommendation. We have changed in Lines 31-32: “We conducted sensitivity and specificity meta-analyses of those studies that used…” to “We conducted meta-analyses of the sensitivity and specificity of those studies that used…”.

**R2.2:** ***"We performed a bivariate random-effects meta-analysis to calculate the pooled sensitivity and specificity with their 95% confidence intervals (95% CI). " Please be very explicit what this overall meta-analyses included. For example one could rephrase as follows "we performed a bivariate random-effects meta-analysis to estimate the pooled sensitivity and specificity with their 95% confidence intervals (95% CI) at each reported common cutoff. For the overall meta-analyses, any GDS-4 version or GDS-5 version separately, by each cut-off and for investigations of heterogeneity, across similar GDS versions by each cutoff".***

**Answer.** Thank you for your observation. We have changed in Lines 34-39: “We performed a bivariate random-effects meta-analysis to calculate the pooled sensitivity and specificity with their 95% confidence intervals (95% CI)” to “We performed bivariate random-effects meta-analyses to calculate the pooled sensitivity and specificity with their 95% confidence intervals (95% CI) at each reported common cut-off. For the overall meta-analyses, we evaluated each GDS-4 version or GDS-5 version separately by each cut-off, and for investigations of heterogeneity, we assessed altogether across similar GDS versions by each cut-off”.

**R2.3:** ***Results***

***Being very explicit about the cutoff and versions will help one understand the results better. For example, the first set of reported results is about all versions of GDS-4 and GDS-5 separately at cutoff>2. This implies the overall meta-analyses at cutoff 2?? and subsequent cutoffs??***

**Answer.** Thank you for your observation. The sentence implies the overall meta-analyses at cut-off 2. It does not imply subsequent cut-offs. We have changed “at a cut-off ≥ 2” to “at a cut-off 2” for better understanding.

In addition, throughout the manuscript, the “≥” sign was eliminated when referring to cut-off points. For example, we have changed “cut-off ≥ 1” to “cut-off 1”.

**R2.4:** ***Conclusion:***

***This conclusion does not accurately reflect the aim of the review which is to estimate accuracy. For example this could be reworded to " We found several GDS-4 and GDS-5 versions that showed great heterogeneity in estimates of sensitivity and specificity, mostly with a low or very low certainty of the evidence"...........***

**Answer.** Thank you for your observation. We have changed in Line 51: “We found several GDS-4 and GDS-5 versions that showed great heterogeneity, mostly with a low or very low certainty of the evidence” to “We found several GDS-4 and GDS-5 versions that showed great heterogeneity in estimates of sensitivity and specificity, mostly with a low or very low certainty of the evidence”

**R2.5:** ***MAIN TEXT***

***Statistical analyses section page 7***

***The overall meta-analyses vs heterogeneity is unclear. For example please see comparisons below:***

***Lines 133-135***

***We conducted meta-analyses of the sensitivity and specificity of GDS-4 and GDS-5 versions whenever studies fulfilled the following condition: 1) There was more than one study that compared the same version of GDS-4 or GDS-5 and used the same cut-off point.***

***Lines 143-144***

***In addition, we meta-analyzed all the included studies that assessed any GDS-4 version, and all the studies that assessed any GDS-5 version, by each cut-off.***

**Answer.** Thank you for your observation. For the overall meta-analyses, we evaluated each GDS-4 version or GDS-5 version separately by each cut-off. Regarding investigations of heterogeneity, we assessed altogether across any GDS-4 version or GDS-5 version by each cut-off.

We have changed Lines 133-135 to “We conducted meta-analyses of the sensitivity and specificity of each of the GDS-4 and GDS-5 versions whenever studies fulfilled the following condition: 1) There was more than one study that compared the same version of GDS-4 or GDS-5 at the same cut-off point”.

We have changed Lines 143-144 to “In addition, we meta-analyzed altogether the results of the included studies that assessed the same cut-off point of any GDS-4 version. Likewise, we meta-analyzed altogether the results of the included studies that assessed the same cut-off point of any GDS-5 version”.
